# The mutational landscape of *MYCN*, *Lin28b* and *ALK*^*F1174L*^ driven murine neuroblastoma mimics human disease

**DOI:** 10.18632/oncotarget.23614

**Published:** 2017-12-22

**Authors:** Bram De Wilde, Anneleen Beckers, Sven Lindner, Althoff Kristina, Katleen De Preter, Pauline Depuydt, Pieter Mestdagh, Tom Sante, Steve Lefever, Falk Hertwig, Zhiyu Peng, Le-Ming Shi, Sangkyun Lee, Elien Vandermarliere, Lennart Martens, Björn Menten, Alexander Schramm, Matthias Fischer, Johannes Schulte, Jo Vandesompele, Frank Speleman

**Affiliations:** ^1^ Center for Medical Genetics, Ghent University, Ghent, Belgium; ^2^ Cancer Research Institute Ghent, Ghent University, Ghent, Belgium; ^3^ Department of Pediatric Oncology and Hematology, University Children’s Hospital, Essen, Germany; ^4^ Department of Experimental Pediatric Oncology, University Children's Hospital of Cologne, Cologne, Germany; ^5^ Center for Molecular Medicine Cologne, University of Cologne, Cologne, Germany; ^6^ BGI-Shenzhen, Bei Shan Industrial Zone, Yantian District, Shenzhen, Guangdong, China; ^7^ Center for Pharmacogenomics and Fudan-Zhangjiang Center for Clinical Genomics, Collaborative Innovation Center for Genetics and Development, School of Life Sciences, Fudan University, Shanghai, China; ^8^ Department of Computer Science, Artificial Intelligence Group, TU Dortmund, Dortmund, Germany; ^9^ Medical Biotechnology Center, VIB, Ghent, Belgium; ^10^ Department of Biochemistry, Ghent University, Ghent, Belgium; ^11^ Pediatric Oncology and Hematology, Charité University Medicine, Berlin, Germany

**Keywords:** neuroblastoma, mouse model, exome sequencing, array CGH

## Abstract

Genetically engineered mouse models have proven to be essential tools for unraveling fundamental aspects of cancer biology and for testing novel therapeutic strategies. To optimally serve these goals, it is essential that the mouse model faithfully recapitulates the human disease. Recently, novel mouse models for neuroblastoma have been developed. Here, we report on the further genomic characterization through exome sequencing and DNA copy number analysis of four of the currently available murine neuroblastoma model systems (*ALK,* Th-*MYCN,* Dbh-*MYCN* and *Lin28b*). The murine tumors revealed a low number of genomic alterations – in keeping with human neuroblastoma - and a positive correlation of the number of genetic lesions with the time to onset of tumor formation was observed. Gene copy number alterations are the hallmark of both murine and human disease and frequently affect syntenic genomic regions. Despite low mutational load, the genes mutated in murine disease were found to be enriched for genes mutated in human disease. Taken together, our study further supports the validity of the tested mouse models for mechanistic and preclinical studies of human neuroblastoma.

## INTRODUCTION

Mouse models are essential tools in the study of the molecular pathogenesis of cancer. They enable, amongst others, the analysis of cooperative genetic events, dynamic regulation of perturbed gene expression during tumor formation and the study of tumor clonality and heterogeneity. Furthermore, facing the emerging challenges of combination treatments of novel drugs, these models now facilitate close alignment of *in vivo* preclinical and early clinical studies [1]. One of the important issues is to determine to what extent the mouse model faithfully recapitulates the human disease and therefore how well it may serve as a preclinical model. Relevant features to study are tumor localization, histology, metastatic pattern as well as genomic features such as RNA expression, DNA copy number alterations and mutation profiles.

For almost two decades, only one transgenic mouse model for neuroblastoma was available (TH-*MYCN*). TH-*MYCN* mice express the human *MYCN* gene under the control of the rat tyrosine hydroxylase (Th) promotor [2], which results in neuroblastic tumors in about 50% of transgenic animals. This model delivered many valuable insights into neuroblastomagenesis [3]. Recently, a novel *MYCN* Cre-inducible transgenic mouse model was established, driving *MYCN* expression from the Dbh promotor integrated into the murine ROSA26 locus (LSL-*MYCN*;*Dbh-i*Cre) [4]. This model has a relatively high penetrance of tumor formation (> 75%). It also overcomes some of the limitations of the TH-*MYCN* mouse model, as tumor localization more closely resembles the human disease. Moreover, two novel *bona fide* neuroblastoma oncogenes have been identified, *ALK* and *LIN28B*, that are sufficient to drive tumor formation when overexpressed in the neural crest or seem to increase the oncogenic potential of MYCN overexpression [5–8].

Whole exome and genome sequencing studies in human primary neuroblastoma provided the first insights into the genome-wide mutational spectrum of this tumor. Overall, similar to findings in other embryonal tumors, mutation frequency was very low with an average of 12 protein coding gene mutations per individual case [9]. *ALK* mutations were detected in approximately 10% of cases, in keeping with previous reports [10]. Pathway analysis of the rare variants in neuroblastomas showed enrichment for genes implicated in neuritogenesis [9]. All other mutations were found at lower frequency except for *ATRX* mutations that were observed in up to one third of cases in older children (> 6 years old) and young adults [11]. *ATRX* was also frequently altered due to inactivating structural changes such as deletions. Taking into account both gene copy number and mutation status, a somewhat higher incidence of alterations of 11% was reported for *ARID1A* and *ARID1B* [12]. Unexpectedly, a similar combination of genomic analyses revealed activating alterations at or near the *TERT* locus in 20% of the high risk cases, almost exclusively *MYCN* non-amplified cases with normal *ATRX* status [13]. Rare, but functionally relevant mutations in *bona fide* cancer genes were also detected such as the *BRAF*^V600E^ and *NRAS*^P44L^ hotspot mutations, hinting at the importance of RAS signaling in a fraction of human neuroblastomas [14]. In relapsed neuroblastoma, up to 78% of cases showed mutations in RAS/MAPK pathway genes, pointing at a role in tumor aggressiveness and therapeutic failure [15, 16].

Here, we describe DNA copy number and whole exome mutation analysis in a total of 36 matched tumor/normal samples from genetically engineered neuroblastoma mouse model systems driven by *MYCN* [2, 4]*, ALK*^F1174L^ [5]*, MYCN/ALK*^F1174L^ or *Lin28b* [6]. Our findings confirm the low incidence of single base pair mutations and reveal recurrent DNA copy number alterations as observed in the human counterpart. Mutation load correlates with the age of onset of tumor formation, with absence of mutations in the most rapidly developing *MYCN/ALK*^F1174L^ and *Lin28b* driven mouse tumors. In one *Th-MYCN* driven tumor, a bi-allelic *Dicer1* missense mutation known to affect the activity of the RNAse III domain was observed, reducing 5p end processing of pre-miRs including reduced levels of miRNAs of the let-7 family. Together with the proven driver role of *LIN28B*, this unique observation further supports the central role for let-7 in neuroblastoma development.

## RESULTS

### Low numbers of mutations revealed by exome sequencing

On average, 86.29 million (stdev 37.84 million) reads per sample mapped to the murine genome. This resulted in an average coverage of 81.62 fold (stdev 35.96, median 62.61) and a total of 85.01% (stdev 6.9%) of coding bases of the murine genome reaching a coverage of > 20 fold. Details per sample are indicated in Supplementary Table 1.

**Figure 1:**
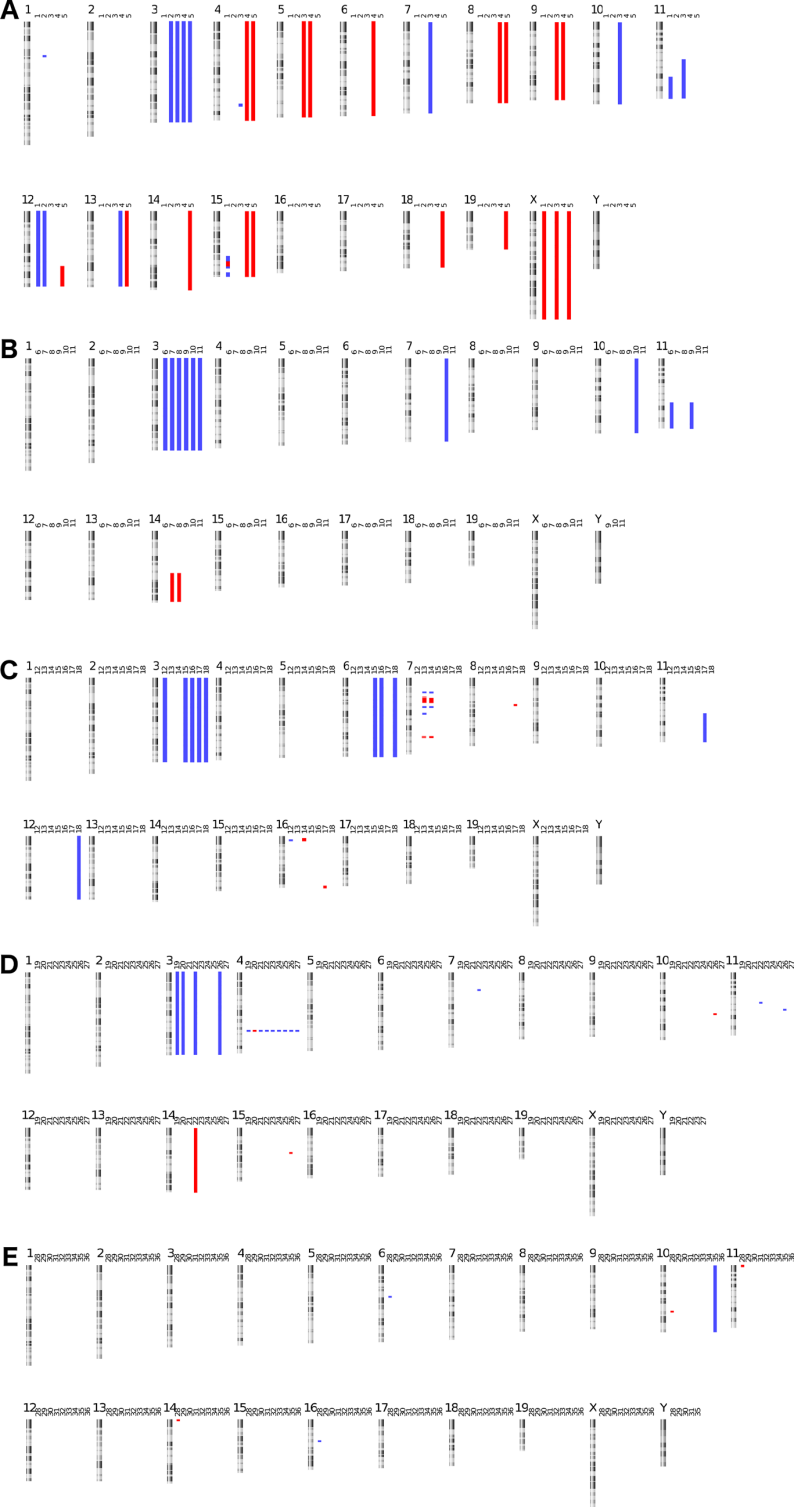
Murine tumor kayo views: DNA copy number profiles of murine tumor samples Gains are indicated in blue, losses in red (**A**. ALK ^F1174L^, **B**. Lin28b, **C**. LSL-MYCN, **D**. Th-MYCN **E**. ALK ^F1174L^/MYCN).

**Figure 2:**
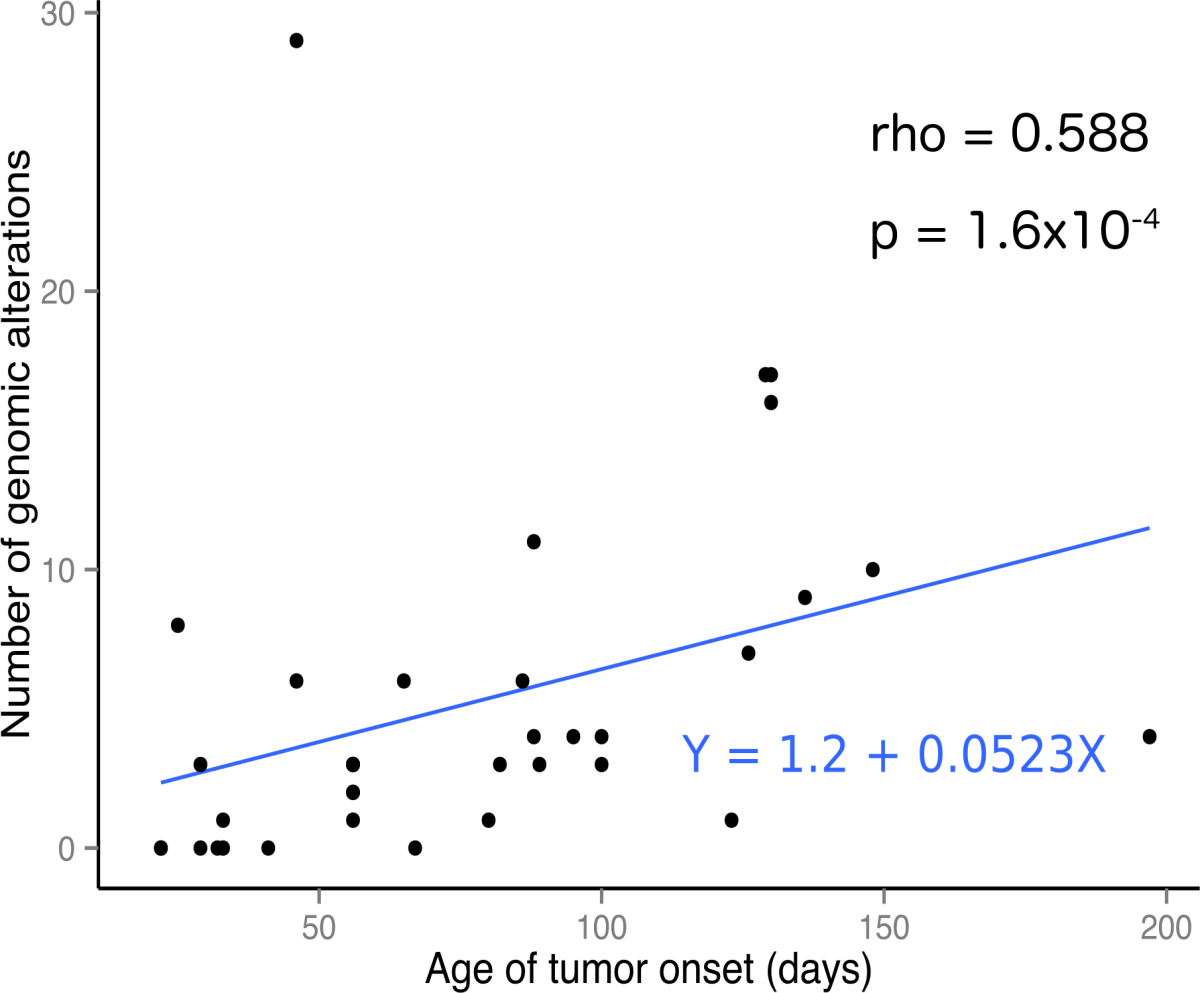
Number of genomic alterations per tumor correlates with tumor age of onset The number of somatic murine genomic alterations plotted against the age of the mouse at tumor onset showing a positive correlation. This correlation is also observed in human tumors [9]. The correlation is not strong but highly significant as indicated by the Spearman’s rank correlation rho and *p* values.

We found an average of 2.87 somatic mutations per exome (range 0–24) with an average of 1.06 (range 0– 10) of these mutations having a possible damaging effect on protein function (defined as non-synonymous coding, frame shifting or truncating mutations, further referred as non-synonymous coding) (see Table 1 for overview). One outlier case was observed with 24 mutations, 21 of which were located in a 45 Mb stretch on chromosome 7, a region heavily affected by copy number changes in this tumor suggesting chromothripsis (see Figure 1C, tumor 13 and 14). The number of somatic mutations positively correlates with the onset of tumor formation (see Figure 2) (rho = 0.588, *p* = 1.6x10^-4^, Spearman’s rank correlation). The Lin28b and Th-*MYCN*-*ALK* driven tumors, which appear earliest, within 60 days after birth, also have the lowest average mutation burden of 0.33 and 0.75 mutations per exome, respectively. The other tumor models have a higher average mutation burden of 3.89 (Th-*MYCN*), 5.5 (Dbh-*MYCN*) and 4.75 (*ALK*) mutations per exome and exhibit a longer average time to tumor formation. Of particular interest is the observation that cases without mutations in the coding genome were observed in each of the mouse models. When also considering DNA copy number changes, all mouse models have either mutations or copy number changes except for one of the Th-*MYCN* tumors (case 27) and all but three of the *MYCN*-*ALK*^F1174L^ double transgenic mice tumors that did not accumulate additional events during tumor development as determined with the methods applied. Supplementary Table 2 contains the non-synonymous coding and Supplementary Table 3 all mutations found in all tumors.

**Table 1:** Genomic alterations in murine tumors

				Array CGH				Somatic exome sequencing	
Sample id	Mouse id	Model	Age of tumor onset	Status	Whole chromosome alterations	Segmental alterations	Number of focal alterations-	number of mutations	number of consequence mutations
4	4	ALK	136	whole chromosome	9 (+3;-4;-5;-6;-8;-9;+13;-15;-X)	-	-	0	0
3	3	ALK	148	segmental	4 (+3;+7;+10;-X)	1 (+11q)	1	4	3
1	1	ALK	130	segmental	2 (+12;-X)	1 (+11q)	3	11	3
5	5	ALK	130	segmental	11 (+3;-4;-5;-8;-9;-13;-14;-15;-18;-19;-X)	1 (-12q)	-	4	1
2	2	ALK	197	focal	2 (+3;+12)	-	2 (including MYCN)	-	-
11	7	LSL-Lin28b	56	whole chromosome	1 (+3)	-	-	0	0
10	7	LSL-Lin28b	56	whole chromosome	3 (+3;+7;+10)	-	-	0	0
9	7	LSL-Lin28b	56	whole chromosome	1 (+3)	1 (+11q)	-	0	0
8	6	LSL-Lin28b	56	segmental	1 (+3)	1 (-14q)	-	1	1
7	6	LSL-Lin28b	56	segmental	1 (+3)	1 (-14q)	-	1	1
6	6	LSL-Lin28b	56	segmental	1 (+3)	1 (+11q)	-	0	0
12	8	LSL-NMYC	89	segmental	1 (+3)	1 (+16p)	1	0	0
13	9	LSL-NMYC	46	focal	-	-	6 ( all on chromosome 7)	0	0
14	9	LSL-NMYC	46	focal	-	-	5 (4 on chromosome 7)	24	10
15	10	LSL-NMYC	100	whole chromosome	2 (+3;+6)	-	1	0	0
16	10	LSL-NMYC	100	whole chromosome	2 (+3;+6)	-	1	1	0
18	12	LSL-NMYC	88	whole chromosome	3 (+3;+6;+12)	-	-	8	1
17	11	LSL-NMYC	88	segmental	1 (+3)	1 (+11q)	2	0	0
24	18	TH-MYCN	86	flat	-	-	0 (skint gene locus)	6	3
20	14	TH-MYCN	129	whole chromosome	1 (+3)	-	0 (skint gene locus)	16	6
23	17	TH-MYCN	65	flat	-	-	0 (skint gene locus)	6	2
19	13	TH-MYCN	123	whole chromosome	1 (+3)	-	0 (skint gene locus)	0	0
25	19	TH-MYCN	82	flat	-	-	0 (skint gene locus)	3	0
21	15	TH-MYCN	67	flat	-	-	0 (skint gene locus)	0	0
22	16	TH-MYCN	95	whole chromosome	2 (+3;-14)	-	2 (+ skint gene locus)	0	0
27	21	TH-MYCN	80	focal	-	-	0 (skint gene locus)	1	1
26	20	TH-MYCN	126	focal	1 (+3)	-	3 (+ skint gene locus)	3	1
28	22	TH-MYCN-ALK	25	focal	-	-	5	3	1
29	23	TH-MYCN-ALK	41	flat	-	-	-	0	0
32	26	TH-MYCN-ALK	29	flat	-	-	-	0	0
31	25	TH-MYCN-ALK	29	flat	-	-	-	3	1
33	27	TH-MYCN-ALK	22	flat	-	-	-	0	0
34	28	TH-MYCN-ALK	22	flat	-	-	-	0	0
35	29	TH-MYCN-ALK	33	whole chromosome	1 (+10)	-	-	0	0
36	30	TH-MYCN-ALK	33	flat	-	-	-	0	0
30	24	TH-MYCN-ALK	32	flat	-	-	-	0	0

The genomic alterations found in the murine tumors divided up into array CGH and exome sequencing results. Array CGH profiles where evaluated as having whole chromosomal copy number changes, segmental (large chromosomal sections) or focal alterations. For the chromosomal and segmental alterations, we indicate the chromosome or region altered, a + means a gain while a – indicates a loss of chromosomal material. For the focal alterations, we list the number of alterations found. In the exome sequencing columns, we list the total number of mutations and the number of mutations predicted to have a consequence at the protein level.

Comparative cross species analysis of all 25 non-synonymous coding mutated genes detected in the mouse models resulted in three matches (*DICER1*, *ZNF574* and *PTCH1*, see Table 2C) in the list of 883 genes reported to harbor non-synonymous coding mutations in human neuroblastoma. Fisher’s exact test (*p* = 0.086, see Table 2A) indicates borderline significant overlap.

Table 2Cross genomics analysis for mutations found in murine tumors

**Table:** A: for all murine tumors

	Murine			
	*p*-val Fisher’s exact test: 0.0865	non-synonymous mutations	not mutated	total
**Human**	non-synonymous mutations	3	880	883
	not mutated	22	20021	20043
	total	25	20901	20926

**Table:** B: for MYCN driven tumors

	Murine			
	*p*-val Fisher’s exact test: 0.0278	non-synonymous mutations	not mutated	total
**Human**	non-synonymous mutations	3	880	883
	not mutated	13	20030	20043
	total	16	20910	20926

**Table:** C: overlapping mutated genes

Murine gene name	Ensembl murine gene ID	Relation	Human gene name	Ensembl Human gene id	Consequence in murine model
Dicer1	ENSMUSG00000041415	ortholog_one2one	DICER1	ENSG00000100697	missense
Zfp574	ENSMUSG00000045252	ortholog_one2one	ZNF574	ENSG00000105732	missense
Ptch1	ENSMUSG00000021466	ortholog_one2one	PTCH1	ENSG00000185920	missense

We explored enrichment of the mutations found in murine tumors for genes known to be mutated in human neuroblastoma. From Table 2A we can see the number of overlapping genes (3) to not be enriched (0.0865 Fisher’s exact test) for genes mutated in human neuroblastoma 883 for a total of 25 genes showing mutations in the murine tumors. If we restrict this analysis to the MYCN driven tumors we do achieve significance (0.0278 Fisher’s exact test) as 3 out of 16 genes mutated in murine tumors show overlap with the genes mutated in human neuroblastoma. In Table 2B we show the murine and human name and ids for the genes showing mutations in both murine and human tumors.

### Recurrent and syntenic DNA copy number alterations observed

All but 10 of the murine tumors harbor copy number alterations as determined by arrayCGH (see Figure 1). The most frequently recurring alteration is a whole chromosome gain of chromosome 3. This specific alteration was previously described in the Th promoter driven MYCN murine model as occurring in up to 40% of the cases but its relevance remains elusive [17]. In our analysis, we confirm this high frequency in *MYCN* (7/15 cases), *ALK* (4/5 of cases) and *Lin28b* (6/6 of cases) driven neuroblastoma models. Murine chromosome 3 is syntenic to several human chromosomes, with the centromeric region of chromosome 3 syntenic with chromosome bands 1p31.2 to 1q32.1. This region is in part overlapping the region that is known to be gained in MYCN amplified neuroblastoma [18].

Of note is the high number of segmental 11q gains across the model systems (5/36 tumors). This region is syntenic to the entire human chromosome 17 that is the most frequently observed gained chromosome in neuroblastoma [18]. An overview of the copy number alterations found in the tumors is given in Table 1.

We did not find any evidence of *Tert* rearrangements as only one whole mouse chromosome 13 gain and one loss were found in 2 tumors. No focal alterations at the murine *Tert* locus at 13qC1 were observed. The resolution of our study however is low for detection of copy number events and copy number neutral events (translocations) are undetectable by array CGH.

### Enrichment of human tumor alterations observed in MYCN driven neuroblastoma model system

The *MYCN* driven murine neuroblastoma tumors all harbor additional genomic alterations. A mean of 5.5 mutations per sample is found in the 15 tumors with a range of 0 to 24 mutations. All but one tumor showed at least one, but mostly multiple whole and/or partial chromosomal copy number alterations. Of particular interest is the large number of whole chromosome 3 gains in 7 out of 15 (46%) cases in this series. Also, a focal region of structural variation (multiple gains and losses) on chromosome 4, not observed in the other mouse models, was present in all Th-*MYCN* driven tumors. As we observed this structural alteration in all tumors, this may be the result of the insertion site of the *MYCN* transgene. This defect has not been described in previous copy number studies of this model system, possibly due to the limited resolution of the BAC clone based microarray of the previous analyses [17]. The location of this focal copy number change hotspot overlaps with the *Skint* gene cluster, a frequent locus for genomic rearrangements.

**Figure 3:**
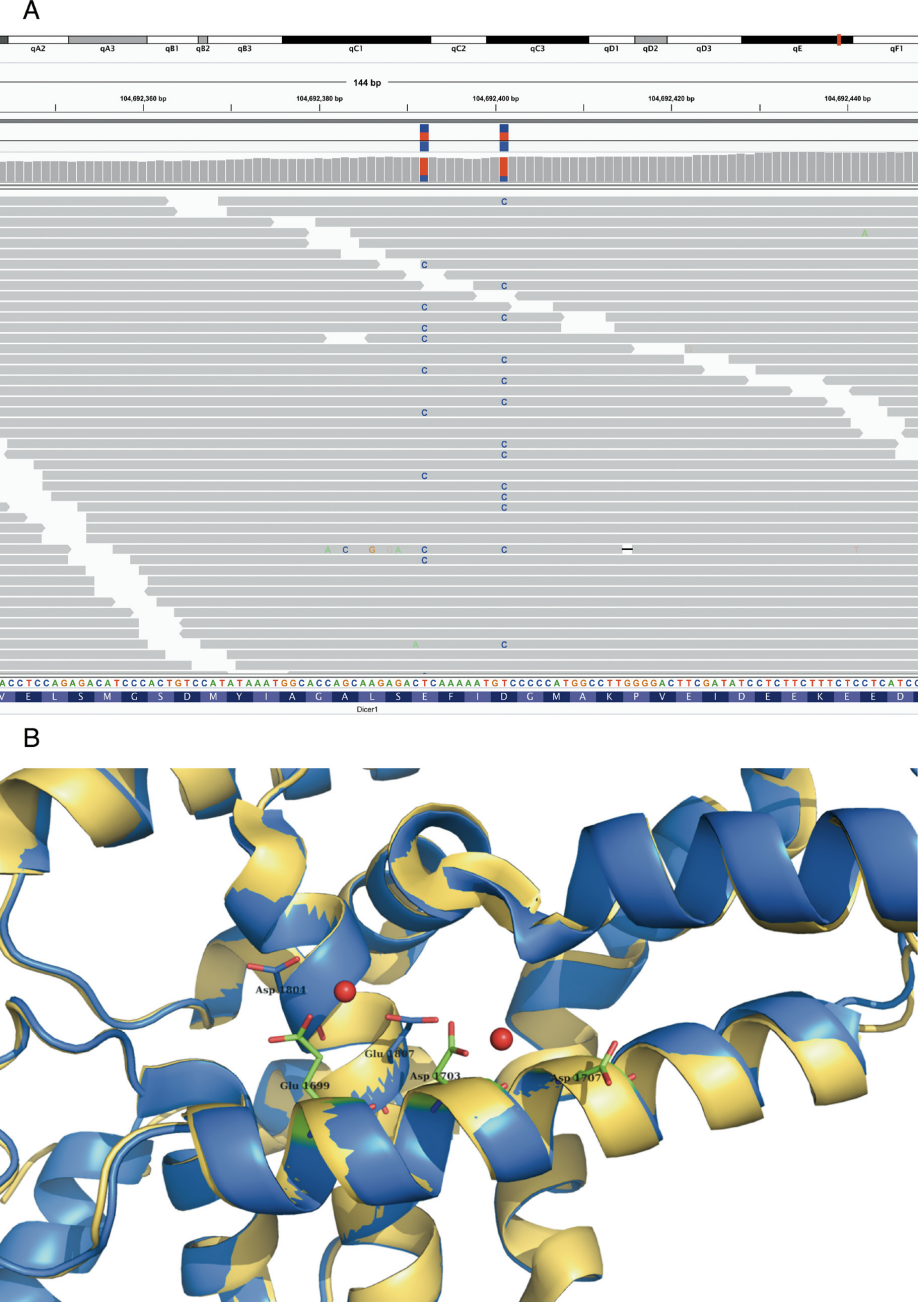
Bi allelic Dicer1 mutation Bi-allelic Dicer1 mutation **A**. IGV view of DICER1 mutated residues in tumour case 20: all but one read (with multiple mismatches and low mapping quality) show only one mismatch which indicates a bi allelic mutation in this tumor. **B**. mutated residues which are shown as sticks (in blue) are located at the surface of the protein, and bind together with Glu1699, Asp1703 and Asp1707 Mg (red balls). PDB-entry 3C4B [58] is shown in blue, PDB-entry 2EB1 [60] is shown in yellow.

Mutations previously reported in human neuroblastoma were enriched in the *MYCN* driven mouse tumors (see Table 2B) (*p*-value of 0.0278, Fisher’s exact test). Of particular interest, a bi-allelic *Dicer1* mutation (see Figure 3) conferring a glycine substitution at position 1804 and 1807 was observed in one single mouse tumor. This mutation is known to cause a loss of negative charge in the magnesium-binding pocket of the RNAse IIIb domain of both the murine *Dicer1* and human *DICER1* protein and has been reported as recurrent mutation in ovarian germ cell tumors. This results in preferential processing of 3p versus 5p miRNAs [19]. We observed this phenotype in the murine tumor that harbors the biallelic dicer mutations by small RNA sequencing. When calculating the average ratio of mature 3p to 5p expression for each pre-miR we observed a value of 1.02 for the *Dicer1* mutant tumor and significantly (z-score 6.71, *p*-value 9.24x10^-12^) lower values of 0.97, 0.95 and 0.95 in three control tumors (two *MYCN* driven, one *Lin28b* driven, all of them not harboring *Dicer1* mutations). The bi-allelic *Dicer1* mutations co-occurred together with 15 other mutations including six non-synonymous coding ones and a whole chromosome 3 gain.

In one Dbh-*MYCN* tumor that harbors whole chromosome gains in chromosomes 3, 6 and 12, we observed a total of eight mutations of which one was a non-synonymous coding mutation. The latter mutation is occurring in the *Ptch1* gene known to play a role in the hedgehog signaling pathway involved in neuroblast differentiation [20]. A p.A300D missense mutation in the *PTCH1* gene was previously reported in human neuroblastoma [12], suggesting that missense mutations could impair neuroblast differentiation and cooperate in *MYCN* driven tumor formation.

The third gene mutated in both murine and human neuroblastoma is *ZNF574* (murine ortholog Zfp574). The Th-*MYCN* driven tumor which harbors this mutation is showing a total of 6 mutations of which 2 are non-synonymous (the second one in an olfactory receptor gene, likely a passenger mutation). Copy number analysis of this tumor reveals only a small number of focal deletions in its genome.

Two tumors of the Dbh-*MYCN* model in this set are derived from the same mouse and are designated “multiple primaries” as they occur in the left and right adrenal glands, which would not be a typical metastatic spread of an adrenal derived tumor. We sequenced both tumors and noticed a similar complex pattern in chromosome 7 copy number changes. Metastatic spread of one primary tumor thus seems a more likely explanation for the multiple tumors observed in this mouse. Of the 24 mutations observed in the left adrenal mass, as many as 19 occurred on chromosome 7, and 17 reside within a single gene (*C2orf78*), located in a region of extensive chromosomal rearrangements. A possible explanation for this is the occurrence of chromothripsis, a complete shattering of a single chromosome also described to occur in primary human neuroblastoma [9, 21].

We have confirmed the occurrence of whole chromosome 6 gains previously reported in this model system with partial synteny to human chromosomes 7p, 7q and 12p [4]. Gains in chromosome 7 have been described to occur frequently in human neuroblastoma.

Of note is that loss of chromosome 1p is frequently reported to co-occur with *MYCN* amplification in human neuroblastoma. The synteny of this human chromosomal region is scattered over many of the murine chromosomes of which large chunks reside on chromosomes 4 and 5. We did not observe any copy number alterations of these two chromosomes in the murine tumors but did find a *Tp73* mutation in one of the *MYCN* driven tumors, this gene is located at the human 1p36 region that is frequently deleted in high-risk neuroblastoma.

### Additional genomic alterations required for ALK ^F1174L^ driven tumor development in murine model system

Activating anaplastic lymphoma kinase (*ALK*) mutations are the most frequent cause of familial neuroblastoma [22]. We sequenced four tumors derived from the *ALK*^F1174L^ transgenic murine model [5]. On top of the DNA copy number changes that mimic the aberrations known to occur in human neuroblastoma, three out of four tumors harbored non-synonymous coding mutations. The relatively late onset of tumor formation (on average 136 days) suggests the requirement of additional oncogenic events in addition to mutant *ALK*. Two tumors show a chromosome 11q gain syntenic to human 17q. One other tumor shows a chromosome 12 gain with an additional focal amplification on that chromosome encompassing the *Mycn* gene locus. One of the 11q gained tumors also contains four mutations of which three are non-synonymous coding. Two of these mutations affect the same allele of the *Cfh* gene, mutated in 340 of 20,238 cases in the COSMIC database among which many lung adenocarcinoma and lung squamous cell carcinomas, a disease with a known role for *ALK* as a tumor driver in a subset of cases. Another tumor with whole chromosomal gains or losses did not harbor any mutations. Of note, efforts to produce more tumors derived from the *ALK* transgenic founder mouse have thus far been unsuccessful. Further, the penetrance of tumor formation of the series of mice in the original publication was only 50%. These observations led us to conduct a detailed search for the insertion site of the *ALK* transgene in the mice forming tumors. Paired-end whole genome sequencing has enabled us to find at least one genomic integration site for the *ALK* vector construct. Using PCR, we confirmed the insertion location to be on chromosome 4 within the *Kif1b* gene, syntenic to the human gene with the same name that maps to the 1p36 chromosomal region, known to be frequently deleted in *MYCN* amplified tumors. Notably, this gene has been proposed as tumor suppressor in neuroblastoma as it drives cells towards apoptosis upon neuronal growth factor withdrawal [23]. Our data thus support a possible tumor suppressor function for this gene and also suggests that other lesions than *ALK* activation may be required to induce tumor formation. It remains to be determined if *Kif1b* activity is compromised in this mouse model and if more than one mutant *ALK* gene copy is integrated in the Kif1b locus or perhaps at other sites.

### Low mutation and copy number alteration numbers in Lin28b and double transgenic mouse neuroblastoma model

Six tumors derived from the *Lin28b* driven model system were included in our analysis. These tumors represent multiple primary tumors derived from two mice (three each). The tumors were isolated from the ganglion ciliare, and the left and right adrenal gland (see Supplementary Table 4) suggesting they are not metastatic spread of a single tumor. Overall, the number of DNA copy number changes and mutations in these tumors is very low, in keeping with the early onset of tumor formation observed in this model system (mean of 56 days in this series). Whole chromosome 3 gain was observed in all tumors. Only two tumors showed a single mutation, one in an olfactory receptor gene (frequent false positive mutation call or passenger mutation) and one in the murine ortholog of the human *PALD1* gene with no known oncogenic function (and no mentions in the COSMIC database). Two of these tumors show a gain of the 11q genomic region, the murine ortholog of human chromosome 17. Another two tumors have a 14q deletion in a region homologous to the human 13q14.3 to 13q33.1 region showing only sporadic gains in human neuroblastoma [18].

A total of eight *ALK*^F1174L^/*MYCN* double transgenic mice tumors were studied. These mice developed tumors at a mean age of 29 days after birth, which is faster than the *MYCN* or the mutant *ALK* driven single transgenic murine models. In keeping with low mutation burden in rapidly developing tumors, these tumors also had with very few genomic alterations. With only two out of eight samples that show three mutations each (of which just a single non-synonymous coding mutation per sample) the average mutation count per sample is only 0.75 and thus much lower than in both the *MYCN* and *ALK* single transgenic model derived tumors. One of the non-synonymous coding gene mutations found in this model system resides in the *Chga* gene. This gene encodes Chromogranin A, a protein co-released from catecholamine containing neurosecretory granules and expressed in sympathetic neuronal cells. This gene has no known tumorigenic or tumor suppressing potential. The second gene found mutated in a tumor derived from this model system has a mutation in the *Sh2d2a* gene. This gene is involved in immune regulation of T-cells. As *Sh2d2a* T-cell deficient mouse seems to have an increased resistance towards tumor formation of B cell lymphoma [24], it is unclear how this mutation might contribute to neuroblastoma tumor formation. The cooperative effect of *MYCN* amplification and *ALK*^F1174L^ mutation seems to be sufficient to drive malignant transformation in neuronal progenitor cells towards neuroblastoma tumors.

### DICER1 RNAse IIIb domain mutation landscape in human neuroblastoma

To further investigate the biallelic *Dicer1* mutation, we Sanger sequenced the *DICER1* RNAse IIIb domain in 155 primary neuroblastoma cases and the frequently mutated catalytic hotspot in an additional 121 cases. Apart from known SNPs, a single E1803D substitution was found. While this is not a *DICER1* hotspot mutation [19, 25], the position is just a few amino acids away from the catalytic site and three cases have been described to be mutated at this position in the COSMIC and TCGA datasets (one colon and two ovarian adenocarcinoma, all E1803K substitutions). In addition, a truncating mutation in *DICER1* was described in a single case of human neuroblastoma analyzed by whole genome sequencing [9]. No other *DICER1* gene mutations have been reported in other neuroblastoma sequencing efforts [11, 12, 14]. Mutation of the catalytic hotspot of the RNAse IIIb domain thus seem to only occur very sporadically in human neuroblastoma. However, it has been shown that *DICER1* can act as a haplo-insufficient tumor suppressor gene [26]. In keeping with this observation, *DICER1* is located in chromosome band 14q32.13, a region which is known to be frequently affected by deletions in neuroblastoma [27, 28] and other tumors. Moreover, in neuroblastoma, *DICER1* and *ARGONAUTE* RNA expression levels have been shown to correlate with survival, with lower expression levels occurring in high-risk tumors [29]. A truncated *DICER1* gene transcript that misses part of the RNAse IIIb domain has been reported in neuroblastoma cells [30]. We performed a pooled re-analysis of a large cohort of 498 primary neuroblastoma RNA sequencing data [31], thus confirming the expression of this transcript in neuroblastoma. However, coverage in this dataset was insufficient to determine a per sample expression level of truncated *DICER1* transcripts. We determined the expression of this specific transcript in our series of primary neuroblastoma cDNA samples by PCR fragment analysis and found its expression to occur in 222 of 338 samples tested (65.6%, see Supplementary Table 5). Of the samples that express truncated *DICER1*, the average relative expression of the truncated isoform to the total *DICER1* level is 9.6% with a range of 2.8 to 39.0%. Expression of this truncated isoform might be an alternative mechanism in which neuroblastoma cells achieve a molecular effect similar to RNAse IIIb domain mutation.

## DISCUSSION

Our study is the first extensive evaluation of genome wide genetic alterations across four of the currently available transgenic murine neuroblastoma models. We provide further support that the tumors that arise in these mice recapitulate well the human disease in terms of genetic alterations. In a previous study, we explored the Th-MYCN model in relation to MYCN and Lin28b driven microRNA regulation [32]. Here, we focus on the occurrence of base pair mutations and DNA copy number alterations and show that the mouse tumors recapitulate the findings in human primary high-risk neuroblastoma. In keeping with sequencing data in human neuroblastoma, the mutation burden in mouse neuroblastoma is low and some tumors even do not show any detectable alterations using the applied methods. Of notice, we observed differences in the number of genomic events that depend on the involved genomic driver. In particular, Lin28b overexpressing tumors exhibited the fewest additional genomic events which points at a very strong oncogenic driver effect. Likewise, the combined ALK^F1174L^ and MYCN targeted overexpression can be sufficient as the sole two genetic events to drive neuroblastoma formation while a higher number of alterations are observed in the ALK^F1174L^ driven cases. This points at an overall weak oncogenic effect of ALK^F1174L^ mutations while differences in penetrance may be related to different mouse background or can be due to transgene integration into a putative tumor suppressor gene which thus increases the penetrance or decreases onset of tumor formation. Our data thus support the previous observation that mutated ALK in itself does not provide sufficient effect to transform sympathetic neuronal progenitors in the given mouse genetic background while potentiating the oncogenic effect of MYCN overexpression as demonstrated by accelerated tumor formation [7, 33]. These findings also underline one of the limitations of most currently available model systems [3] where uncertainty exists on the insertion location of the transgenic vector. This limitation is addressed in the LSL-*MYCN;Dbh-iCre* [4] model system where transgene integration in the ROSA26 locus is ensured.

As reported before, whole chromosome 3 gains are a remarkably frequent recurring event in MYCN (46% of cases in our series, up to 40% of published cases [17]), ALK (80% of cases) and Lin28b (100% of cases) driven murine neuroblastoma tumors. Interestingly, chromosome 3 gain has not been observed in a BRCA1 driven murine breast cancer model [34], nor a KRAS driven model of non–small cell lung cancer [35] while frequent partial gain of this chromosome was described in a MYC driven murine model of lymphoma [36]. This might point at the importance of chromosome 3 gain in MYC(N) driven oncogenesis.

Our current understanding of the oncogenic role of LIN28B in neuroblastoma tumor formation is that it is a negative regulator of the let-7 family of miRNAs that directly target MYCN [6]. Increased Lin28b activity thus contributes to oncogenic MYCN pathway activity. Observing the same copy number gains as found in the MYCN driven model system (and other MYC driven murine cancer model systems [36]) is a strong indicator of the role of murine chromosome 3 gene dosage contribution to MYCN driven oncogenicity. The short duration until tumor formation, the development of multiple primary tumors per mouse and the low number of copy number changes and mutations found in the tumors indicate that transgenic Lin28b overexpression results in a rapidly developing highly penetrant murine tumor model requiring little if any additional genomic alterations, in keeping with the situation in human atypical teratoid rhabdoid tumors [37].

Several of the non-synonymously mutated genes found are of interest in neuroblastoma tumor formation. Both DCLRE1A and TP73 are involved in DNA repair processes. Other genes involved in these processes have been described to be mutated sporadically in neuroblastoma tumors like the FANCM, FAN1, BRIP1 and MLH1 genes [9, 14]. The mutation of the double strand break repair gene DCLRE1A is of particular interest as impairment of the non-homologous end joining process is believed to be the cause of the characteristic copy number changes in neuroblastoma. Of note, the human neuroblastoma showing the DCLRE1A mutation is also harboring a complex chromosomal alteration on chromosome 7, with multiple copy number changes [38]. TP73 on the other hand is part of the p53 family of transcription factors involved in response to DNA damage and cell cycle arrest. It is one of the highest ranking tumor suppressor candidate genes located on the chromosome 1p36 genomic region showing frequent deletions in neuroblastoma [39].

Defects in neuritogenesis are proposed as one of the mechanisms involved in neuroblastoma tumor formation [9]. The only supporting evidence we found in murine tumors is a mutation in the PTCH1 gene associated to neural tube formation. As an activator of the hedgehog signaling pathway, it plays a role in neuronal differentiation [40, 41]. Defects in neuritogenesis probably are early events in neuroblastoma tumor formation.

ASXL1, a gene known to be associated with poor prognosis in myeloid malignancies when mutated [42] is a ligand binding co-activator of the retinoic acid receptor [43]. Retinoic acid has been used as one of the standard treatments of neuroblastoma as it induces neuronal differentiation. ASXL1 is working in a complex with EZH2 and SUZ12 as part of the PRC2 complex. It reduces the number of H3K27me3 histone marks, resulting in increased RAS driven transcription [44], a pathway proven to be important in neuroblastoma relapse and chemoresistance. Chromatin remodeling genes are recurrently mutated in human neuroblastoma [9, 11, 12, 14].

The occurrence of a bi-allelic hotspot mutation at the catalytically important residues in Dicer1 and the subsequent reduced processing of 5p derived miRNAs documented by small RNA sequencing is strongly suggestive for a functional cancer-driving role in the affected tumor. Similar mutations in orthologous residues of human DICER1 gene have proven biological consequences for miRNA processing and are known to be oncogenic [19, 25]. These mutations occur in various other human embryonic tumors like Wilms tumor [45], non-epithelial forms of ovarian cancer and embryonic rhabdomyosarcoma [46]. The neuroblastoma driving potential of LIN28B and the occurrence of a bi-allelic Dicer1 RNAse IIIb domain mutation is indicative of the importance of miRNA deregulation in neuroblastoma tumor formation. miRNAs are of crucial importance in normal embryonic development and an increasing number of embryonic tumor types are known to have alterations in the miRNA processing machinery [45, 46]. Although they do occur, our data clearly shows that DICER1 mutations are not a major mechanism of miRNA biogenesis alteration in neuroblastoma. A possible alternative mechanism for neuroblastoma cells to achieve the same biological effect would be to express a truncated DICER1 transcript as previously described [30, 47] and documented in this study to occur at substantial rates in primary neuroblastoma. Whether this transcript is neuroblastoma specific and really is contributing to neuroblastoma oncogenesis remains to be explored.

Despite the enrichment of genes mutated in human neuroblastoma in the MYCN driven model systems we studied, we did not find any mutations in some important recurrently altered genes involved in neuroblastoma biology. Mutations and structural alterations involved in genes regulating telomere maintenance are an important emerging theme in neuroblastoma biology. We found no evidence in murine tumors of either *ARID1A* [12] or *B*, *ATRX* [11] or *TERT* [13, 48] mutations. However, in human neuroblastoma TERT rearrangements rarely, if ever, co-occur with MYCN or ATRX alterations [13, 48]. Moreover, the methods we applied are not appropriate to detect copy number neutral structural variations known to cause TERT alterations in human neuroblastoma. Another theme that seems to be under represented in murine tumors are defects in neuritrogenesis genes like *PTPRD, ODZ3* or *CSMD1* [9]. Alterations in these genes possibly are early events in human neuroblastoma development and thus are not required for tumor formation in neuroblastoma model systems overexpressing a strong tumorigenic driver in the neural crest. Overall the low number of recurrent mutations in both human and murine neuroblastoma are indicative of this disease’s genetic heterogeneity.

An important limitation of currently available neuroblastoma murine model systems is that none of them seem to adequately represent the metastatic pattern of human disease. In our murine dataset, we have not found any mutations in genes published to be associated with metastatic disease like *TRKB, NM23, C-MYB, SLUG1, DKK1, NCAM* of integrins and selectins [3].

In conclusion, our study further contributes to the characterization of four of the currently available genetic neuroblastoma mouse models and further supports their validity for preclinical studies. Following this analysis, further follow up should include RNA sequencing, whole genome sequencing and epigenetic profiling in order to further characterize the mouse neuroblastoma genome in more depth as prelude to currently foreseen drug testing to dissect the biological effects of novel treatments in mouse and human tumors.

## MATERIALS AND METHODS

### Mouse model tumors

Tumors were obtained from LSL-*Lin28b; Dbh-iCre* (*n* = 6) [6]; LSL-*MYCN;Dbh-iCre* (*n* = 7) [4], *Th-MYCN* (*n* = 9) [2], *ALK*^F1174L^; *Dbh*-i*Cre* (*n* = 5) [5] and *Th-MYCN*; *ALK*^F1174L^; *Dbh*-*iCre (n* = 9) [5] model systems. Heterozygous state for the transgene of all mice included in this study was ensured by cross breeding with wild type mice. Tumor characteristics are listed in Supplementary Table 4.

### Human neuroblastoma tumors

The samples for DICER1 DNA mutation analysis were available from the Neuroblastoma Research Consortium, a European collaboration of national coordinating neuroblastoma research labs in Ghent, Belgium; Amsterdam, The Netherlands; Essen, Germany; Genova, Italy; Valencia, Spain and Dublin, Ireland. The patient characteristics are given in Supplementary Table 6.

### Exome sequencing and mutation calling

Murine tumor and constitutional DNA was captured using the Agilent SureSelect XT target enrichment system for Illumina paired end sequencing. A total of 200 ng of input DNA was used per sample. DNA was sheared using a Covaris S-series Single Tube Sample Preparation System to a target length of 200 bp (duty cycle 10% intensity 5; 200 cycles per burst for 200 seconds). Subsequently, the DNA was purified using Agencourt AMPure beads and analyzed on an Agilent Bioanalyzer using the high sensitivity DNA assay. After end repair and adapter ligation, performed as specified by the manufacturer’s protocol, the samples were purified with Ampure Beads and then amplified for 6 cycles using Phusion High fidelity PCR reagents. Libraries were sequenced on an Illumina HiSeq 2000 in 2x 100 bp mode.

Raw sequencing data was demultiplexed on the HiSeq instrument using the manufacturer’s software. Mapping was performed to build 37 of the murine reference genome (Genome reference consortium MGSCm37) using BWA [49] (v. 0.5.9). Reads were quality recalibrated using the Genome analysis toolkit [50] (v. 1.6-13-g91f02df) and duplicate reads were removed using Picard tools (v. 1.59). Variants were called using the Genome Analysis Toolkit (GATK) unified genotyper [50] (v 1.6-13-g91f02df). Variants were annotated and sample calls between tumors and controls were compared using our custom cloud based analysis platform seqplorer (https://brenner.ugent.be/seqplorer/) (De Wilde et al., in preparation). Mutations were found by considering the raw read counts in the tumor and matching normal sample. Fisher’s exact test was calculated on the raw read counts for each variant called by the GATK in the tumor sample and subsequently multiple testing corrected (according to Benjamini Hochberg [51]). A mutation was considered if its p-value was significant at the 0.05 level, the percentage of variant reads in the normal sample was under 5% and the percentage of variant reads in the tumor sample was at least 10% higher.

Coverage data was extracted for each sample using the samtools depth option. To evaluate capture efficiency, we defined the target region as the coding parts of the canonical transcript from of all coding genes in the murine genome, according to the Ensembl database (release 68). The total target region comprised of 196,710 coding genomic elements, including 35,131,573 base pairs.

### Whole genome sequencing and transgene integration mapping

To find the genomic insert of the mutant ALK transgene in the murine genome, we sequenced tail derived DNA from mouse 5 included in this analysis. DNA was sequenced as described above, omitting the exome capture step. The reference genome was constructed from the MGSCm37 genome, adding the fasta sequence of the ALK transgene vector as a separate chromosome. We generated a total of 167 million reads which, after mapping with stampy [52] (version 1.0.13), resulted in a genome wide coverage of 8.65 fold with a local coverage of the transgene of 18.34 fold . Subsequently SVDetect [53] (version 0.7) was used in interchromosomal rearrangement mode to identify the integration site of the ALK transgene vector.

### Cross species genomics analysis

To evaluate the overlap of the genes mutated in murine or neuroblastoma disease, we performed a cross genomics analysis using the Ensembl cross genomics API [54] (build 68). For each gene mutated in murine neuroblastoma the Ensembl gene id of the orthologous human gene was retrieved. We then searched for overlap between this gene list and genes mutated in published exome or whole genome sequencing efforts in human primary neuroblastoma. As all of the published gene lists are restricted to mutations that likely affect the protein (non-synonymous coding, frameshift, splice site or premature stop codon), we calculated the enrichment score with a chi squared statistic for the same type of mutations in the murine dataset. Of the 940 genes reported as mutated in human neuroblastoma [9, 11, 12], we were able to obtain an Ensembl database identifier for 883 of them. A total of 20,926 gene pairs were found to be orthologous in human and mouse.

### Array comparative genome hybridization CNV analysis

DNA was isolated using the DNeasy Blood & Tissue Kit (Qiagen) according to the manufacturer’s instructions. ArrayCGH was performed using a 180K (AMADID 027411) mouse whole-genome arrays (Agilent Technologies). Random primed labeling (BioPrime ArrayCGH Genomic Labeling System, Invitrogen) was used to label 400 ng of tumor DNA and matched control DNA with Cy3 and Cy5 dyes (Perkin Elmer), respectively. Hybridization and washing were performed according to the manufacturer (Agilent Technologies). Fluorescence intensities were measured on an Agilent G2505C scanner. Data were extracted using the Feature Extraction v10.1.1.1 software (Agilent Technologies), and further processed with ViVar [55]. Gains and losses were determined using the circular binary segmentation algorithm [56].

### Variant confirmation using sanger sequencing

The mutations found in exome sequencing with a predicted effect on protein were confirmed by Sanger sequencing whenever primer design was successful. Primers were designed for the affected exons involved using the primerXL web tool (http://www.primerxl.org). 10 ng of tumor genomic DNA was amplified in a 25 µl PCR reaction using 250 nM of each primer and Bio-Rad SsoAdvanced mastermix according to the manufacturers protocol. PCR amplicons were Sanger sequenced on a ABI Prism 3100 genetic analyser (Applied Biosystems) according to manufacturer’s protocol. Trace files were interpreted using 4Peaks software (v1.8 http://nucleobytes.com/4peaks/) and aligned to the genome using the UCSC Blat function [57].

### Small RNA sequencing

Small RNA libraries were prepared using the TruSeq small RNA library prep kit (Illumina) and sequenced on a MiSeq instrument (Illumina). Reads were filtered based on quality, trimmed and collapsed using the fastx toolkit (v. 0.0.13). Collapsed reads were mapped to build 38 of the murine reference genome (Genome reference consortium MGSCv38) using bowtie (v. 0.12.7) and annotated using miRBase.

### 3D modeling of DICER1 mutation

The available structures for DICER1 (PDB-entries 3C4B and 3C4T [58] were analysed and compared using Pymol (The PyMol Molecular Graphics System, version 1.7.4 Schrödinger, LLC).

### DICER1 mutation analysis

The RNAse IIIb domain of the DICER1 gene was Sanger sequenced in a series of human primary neuroblastoma samples. For PCR amplification, pxlence PCR assays were used (http://www.pxlence.com). 10 ng of Phi29 amplified DNA (GE Healthcare illustra genomiphi v2 kit) was amplified in 25 µl PCR reaction using 250 nM of each primer and Bio-Rad SsoAdvanced mastermix according to the manufacturer’s protocol. PCR amplicons were Sanger sequenced by Genewhiz (NY, USA). Electropherograms were interpreted using the SeqPilot v.4.2.1 (JSI Medical Systems) software.

### Truncated DICER1 expression profiling

To detect the expression of DICER1 RNAse IIIb lacking isoforms we performed fragment analysis of PCR amplicons generated with cDNA primers spanning the alternative splicing sites of full length and truncated DICER1 as described previously [30]. Fragments were amplified on a LC480 instrument (Roche) using the SsoAdvanced mastermix (Bio-Rad) in 8 µl reaction volume with 250 nM of primer concentration. Amplification was performed with 35 cycles of 95°C for 30 seconds, 55°C for 30 seconds and 72°C for 40 seconds. Amplicons were sized and quantified on a Caliper GX capillary electrophoresis system (Perkin Elmer). The molar ratio of truncated DICER1 expression over full length DICER1 expression was determined using the instrument software.

### Statistics

Statistical analysis and plotting of data was done using the R language and environment for statistical computing (R version 2.15.1; www.R-project.org). Plots were generated using the ggplot2 package [59]. Throughout the manuscript, statistical significance was defined as a *p*-value lower than or equal to 0.05.

## SUPPLEMENTARY MATERIALS TABLES













## Abbreviations

RNA: ribonucleic acid
DNA: deoxyribonucleic acid
BWA: Burrows-Wheeler aligner
GATK: genome analysis toolkit
GRC: genome reference consortium
CGH: comparative genomic hybridization
UCSC: University of California Santa Cruz
