# Supplementary material for: The mutational landscape of MYCN, Lin28b and ALKF1174L driven murine neuroblastoma mimics human disease
Source: Oncotarget. 2017 Dec 22;9(9):8334–49. doi: 10.18632/oncotarget.23614 (PMC5823580; doi:10.18632/oncotarget.23614)
Supplement: Supplementary file 1 [file oncotarget-09-8334-s001.pdf]

The mutational landscape of *MYCN*, *Lin28b* and *ALK*^{F1174L} driven murine neuroblastoma mimics human disease

SUPPLEMENTARY MATERIALS

Supplementary Table 1: Coverage statistics. See_Supplementary_Table 1

Supplementary Table 2: Non synonymous coding mutations in murine tumors. See_Supplementary_Table 2

Supplementary Table 3: Somatic mutations in murine tumors. See_Supplementary_Table 3

Supplementary Table 4: Murine tumor characteristics. See_Supplementary_Table 4

Supplementary Table 5: t-dicer expression ratio. See_Supplementary_Table 5

Supplementary Table 6: Human neuroblastoma sample characteristics.

	**INSS stage**	
1	31	11.23%
2	26	9.42%
3	28	10.14%
4	89	32.25%
4S	19	6.88%
	**Age groups**	
less than 1 year	69	25.00%
over 1 year	124	44.93%
	**Age at diagnosis distribution (in months)**	
minimum	0	
1rst quartile	6	
median	18	
3rd quartile	34	
maximum	163	
	**MYCN status**	
amplified	38	13.77%
non amplified	154	55.80%
	**Array CGH profile subtype**	
1	71	25.72%
2a	45	16.30%
2b	30	10.87%
